# Exploratory Transcriptomic, Genomic, Pharmacogenomic, and Cellular Evaluation of FKBP4 in Lung Adenocarcinoma

**DOI:** 10.3390/biology15181661

**Published:** 2026-09-19

**Authors:** Fangyu Wang, Pengfei Lyu, Weimin Chen, Huiquan Gu, Wenlong Han, Yaolong Yu, Chen Chen, Rui He, Qiang Liu

**Affiliations:** 1 Key Laboratory of Tropical Translational Medicine of the Ministry of Education, School of Basic Medical Sciences, Hainan Medical University, Haikou 571199, China; 2 School of Medicine and Health, Guangzhou City Construction College, Guangzhou 510925, China; 3 Department of Breast-Thoracic Tumor Surgery, Affiliated Nanhua Hospital, University of South China, Hengyang 421009, China; 4 Department of Pharmacology, School of Basic Medical Sciences, Hainan Medical University, Haikou 571199, China; 5 School of Pharmacy, Zhejiang Chinese Medical University, Hangzhou 310053, China; 6 School of Biomedical Sciences, The University of Queensland, Brisbane, QLD 4072, Australia; chen.chen@uq.edu.au

**Keywords:** FKBP4, lung adenocarcinoma, transcriptomics, pharmacogenomics, H1299 cells

## Abstract

FK506-binding protein 4 (FKBP4), also known as FKBP52, is an HSP90 co-chaperone associated with poor outcomes in lung adenocarcinoma (LUAD). We evaluated FKBP4 using TCGA-LUAD, cBioPortal, CellMiner, and H1299 cell data. Among 535 primary tumors, the 20 displayed *FKBP4* correlations from each of the HALLMARK_APOPTOSIS and HALLMARK_PEROXISOME sets remained significant after set-wise Bonferroni correction. cBioPortal identified two *FKBP4* coding alterations among 511 tumors (0.39%), without a recurrent hotspot. No CellMiner association remained significant after Benjamini–Hochberg correction across 263 activity profiles. Three siRNAs reduced *FKBP4* transcript abundance in an archived qPCR screen, although independent transfections could not be confirmed. Only si-FKBP4-3 was assessed by CCK-8, and FKBP4 protein depletion was not confirmed during the assay window. In one archived experiment, mean OD450 values were higher at 48 and 72 h across five technical wells. Because CCK-8 reflects WST-8 reduction, this observation does not establish proliferation, survival, or another defined phenotype. The study therefore identifies tumor-level co-expression associations and a preliminary cellular observation requiring independent and orthogonal validation.

## Simple Summary

FKBP4 is an HSP90 co-chaperone associated with poor outcomes in lung adenocarcinoma. We combined tumor datasets, pharmacogenomic data, and H1299 cell experiments to examine additional FKBP4-related features. The displayed *FKBP4* correlations with apoptosis- and peroxisome-related genes remained significant after set-wise correction. Coding alterations were rare, and no CellMiner drug association remained significant after multiple-testing correction. In one archived H1299 CCK-8 experiment, si-FKBP4-3 produced higher mean OD450 values at 48 and 72 h. Five wells were technical replicates, only one siRNA was tested, and FKBP4 protein depletion was not confirmed during the assay window. CCK-8 alone cannot assign a defined cellular phenotype. This observation is preliminary and hypothesis-generating.

## 1. Introduction

Lung cancer remains the leading cause of cancer mortality worldwide [1]. Lung adenocarcinoma (LUAD) is the main non-small-cell lung cancer subtype [2,3]. Targeted therapies and immune-checkpoint inhibitors benefit selected patients. However, heterogeneity and adaptive resistance still limit durable responses [3,4]. Biomarkers reflecting these processes may improve biological stratification.

FK506-binding protein 4 (FKBP4), also called FKBP52, is a large immunophilin. Its tetratricopeptide-repeat domains bind HSP90 and support client maturation [5,6]. FKBP4 also regulates steroid-receptor trafficking and protein-complex assembly [6,7]. These functions depend on client availability and cellular state.

FKBP4 supports cancer progression in breast and prostate models [8,9,10]. In LUAD, *FKBP4* promotes malignant phenotypes in PC9 and H1975 cells [11]. Our previous TCGA-LUAD study linked high *FKBP4* expression with poor prognosis [12]. That work focused on prognosis, pathway enrichment, and immune associations. The present study examines separate endpoints not reported in that analysis.

Patient tumors and cultured cells capture different levels of FKBP4 biology. Tumors include malignant, stromal, and immune compartments, whereas cell culture isolates a defined background [4]. Considering these levels separately helps distinguish population-level associations from model-specific cellular responses.

We examined *FKBP4* correlations within two MSigDB Hallmark gene sets and assessed coding alterations. We also analyzed NCI-60 drug-response data and three *FKBP4*-targeting siRNAs in H1299 cells. This exploratory, hypothesis-generating study evaluates tumor-level associations alongside a preliminary cellular observation. It does not assign a causal FKBP4 mechanism or a defined cellular phenotype.

## 2. Materials and Methods

### 2.1. TCGA-LUAD Data and Targeted Hallmark Correlation Analysis

TCGA-LUAD RNA-sequencing data were obtained from the Genomic Data Commons using TCGAbiolinks [13,14] (https://bioconductor.org/packages/TCGAbiolinks/, accessed on 16 September 2026). The dataset contained 535 primary tumors and 59 non-tumor samples; only primary tumors entered correlation analyses. This design assessed inter-tumor expression covariation rather than tumor-versus-normal differences.

HTSeq-FPKM values were converted to TPM within each sample using the following equation:

TPM_i_ = FPKM_i_/(Σ_j_ FPKM_j_) × 10^6^

For downstream analyses, TPM values were transformed as log_2_(TPM + 1).

Gene sets were obtained from MSigDB v7.2: HALLMARK_APOPTOSIS (M5902; 161 genes) and HALLMARK_PEROXISOME (M5949; 104 genes) [15,16]. Spearman correlations with *FKBP4* were evaluated across 535 primary tumors. Genes were ranked by absolute Spearman coefficient. The top 20 genes from each set were displayed. Bonferroni correction was applied separately within each gene set. Supplementary Table S1 reports all coefficients, nominal *p*-values, adjusted *p*-values, and within-set ranks. The two sets were retained from the archived analysis.

### 2.2. Genetic Alteration and Human Protein Atlas Review

*FKBP4* coding alterations were queried in cBioPortal using the TCGA-LUAD PanCancer Atlas mutation profile [17,18] (https://www.cbioportal.org/study?id=luad_tcga_pan_can_atlas_2018, accessed on 16 September 2026). This profile contained 511 tumors with mutation data and used precomputed somatic variant calls; no de novo variant calling was performed. No additional custom variant filtering was applied beyond restricting the review to coding alterations reported in the selected cBioPortal mutation profile. Reported coding alterations were reviewed descriptively. Representative HPA images were reviewed for FKBP4 (ENSG00000004478-*FKBP4*) [19,20] (https://www.proteinatlas.org/ENSG00000004478-FKBP4, accessed on 16 September 2026). Selection followed the displayed antibody, cell-line, tissue, and sample annotations. Original HPA scale indicators were retained. The tissue images were unpaired and were not quantitatively compared.

### 2.3. CellMiner Pharmacogenomic Analysis

*FKBP4* expression and NCI-60 drug-response data came from preserved CellMiner matrices [21,22] (https://discover.nci.nih.gov/cellminer/, accessed on 16 September 2026). The dataset contained 263 activity profiles representing 202 unique compound names. Pairwise-complete Spearman correlations linked *FKBP4* expression with each activity profile. Two-sided *p*-values were adjusted across all 263 profiles using the Benjamini–Hochberg method; adjusted *p* < 0.05 defined screen-level significance. Dataset S2 provides the complete analysis, including all seven nominal profiles.

### 2.4. Cell Culture

NCI-H1299 human NSCLC cells were purchased from Immocell Biotechnology (Xiamen, China; IM-H242). The supplier reported ATCC origin and short-tandem- repeat authentication. Archived records did not document in-house re-authentication or mycoplasma testing. Cells were maintained in RPMI-1640 (Solarbio, Beijing, China) containing 10% fetal bovine serum (Cellmax, Beijing, China) and 1% penicillin–streptomycin (Solarbio, Beijing, China) at 37 °C with 5% CO_2_.

### 2.5. siRNA Transfection

Guangzhou IGE Biotechnology (Guangzhou, China) synthesized three *FKBP4*-targeting siRNAs and a non-targeting control (si-NC). H1299 cells received 50 nM siRNA using Lipofectamine 3000 (Invitrogen/Thermo Fisher Scientific; Carlsbad, CA, USA; L3000001). Transfection followed the manufacturer’s instructions, and total RNA was collected after 24 h. Table 1 reproduces the supplied sequences exactly, including lowercase 3′-terminal “tt” residues. The records did not specify whether these residues represented overhangs or chemical modifications. Knockdown estimates were normalized to si-NC. The qPCR screen also included an untreated control (CK).

### 2.6. RNA Extraction and RT-qPCR

Total RNA was extracted using TRIzol reagent (Invitrogen, Carlsbad, CA, USA), and one microgram underwent reverse transcription. RT-qPCR used SYBR Green chemistry (Applied Biosystems, Thermo Fisher Scientific, Waltham, MA, USA) in 10 µL reactions. Each reaction contained 5 µL master mix, 0.2 µL of each primer, 1 µL cDNA, and 3.6 µL nuclease-free water. Cycling started at 95 °C for 30 s, followed by 40 cycles at 95 °C for 5 s and 60 °C for 30 s. Available records documented three replicate samples per condition, each measured in duplicate qPCR wells; separate transfections could not be confirmed. *GAPDH* served as the reference gene.

Relative *FKBP4* expression was derived from 2^−ΔCq^ values within the comparative-Cq framework [23]. Replicate-level values were normalized to the arithmetic mean of the si-NC replicates. Primer sequences are listed in Table 2.

### 2.7. CCK-8 Assessment

H1299 cells were transfected with si-FKBP4-3 or si-NC and seeded in 96-well plates at approximately 2000–5000 cells per well. Five technical wells were used for each condition and time point. CCK-8 reagent (10 µL) was added at 24, 48, 72, and 96 h. After 1–2 h at 37 °C, absorbance was measured at 450 nm. The corresponding blank-well mean was subtracted from each reading. The archived CCK-8 dataset contained si-NC and si-FKBP4-3 conditions only and did not include a separate mock-transfection condition. Archived records did not include FKBP4 protein measurements during the CCK-8 assay window, a second siRNA phenotype, an siRNA pool, rescue experiments, or orthogonal functional assays.

### 2.8. Statistical Analysis

TCGA analyses used R version 4.0.2, with Bonferroni adjustment within each Hallmark set. CellMiner correlations used pairwise-complete observations and two-sided *p*-values. Benjamini–Hochberg correction was applied across 263 activity profiles. Cell-based results are summarized as mean ± SD. qPCR values represent three replicate samples, each measured in duplicate wells. CCK-8 values represent five technical wells from one archived experiment. Because independent biological replication could not be verified, no inferential *p*-values were reported for either cell-based assay. Accordingly, between-experiment reproducibility could not be estimated from the archived cell-based data.

## 3. Results

### 3.1. FKBP4 Shows Bidirectional Associations with Apoptosis- and Peroxisome-Related Hallmark Genes

**Figure 1:**
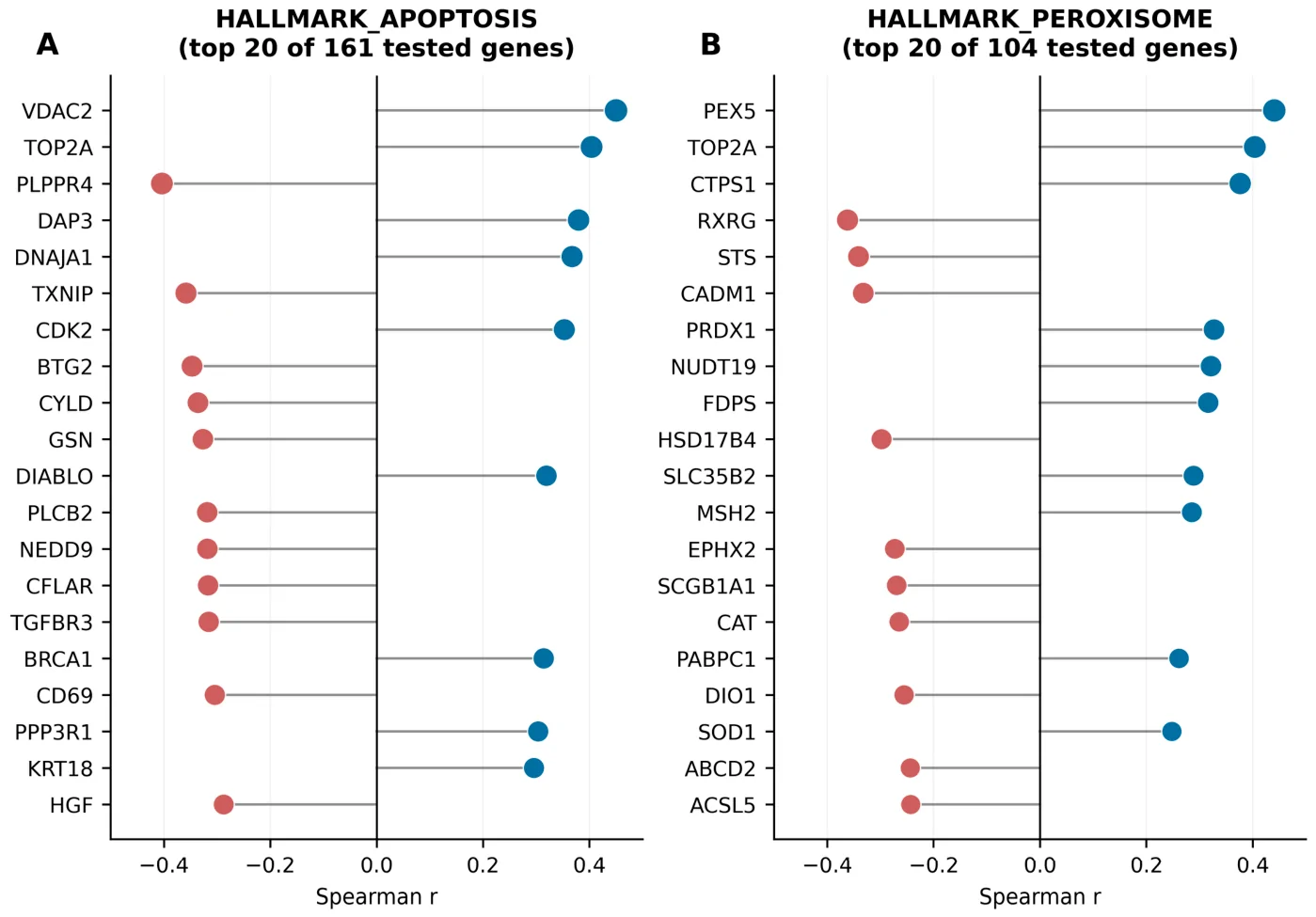
FKBP4 correlations with MSigDB Hallmark genes in 535 TCGA-LUAD primary tumors. (**A**) Twenty genes with the largest absolute Spearman coefficients in HALLMARK_APOPTOSIS. (**B**) Twenty genes with the largest absolute coefficients in HALLMARK_PEROXISOME. Dot position indicates Spearman r, and dot size indicates |r|. Blue circles indicate positive correlations, whereas red circles indicate negative correlations. Horizontal segments connect each estimate to zero. All displayed correlations passed set-wise Bonferroni correction. Full results are provided in Supplementary Table S1.

Within HALLMARK_APOPTOSIS, *FKBP4* showed positive and negative correlations across the 535 tumors (Figure 1A). The largest coefficients included *VDAC2* (r = 0.450), *TOP2A* (r = 0.404), *PLPPR4* (r = −0.404), and *DAP3* (r = 0.380). All 20 displayed genes passed the set-wise Bonferroni threshold, with complete results provided in Supplementary Table S1.

HALLMARK_PEROXISOME showed a similar bidirectional correlation pattern (Figure 1B). The largest coefficients included *PEX5* (r = 0.441), *TOP2A* (r = 0.404), *CTPS1* (r = 0.377), and *RXRG* (r = −0.362). All 20 displayed genes remained significant after set-wise Bonferroni correction.

### 3.2. FKBP4 Shows Sparse Coding Alterations and Descriptive HPA Localization

**Figure 2:**
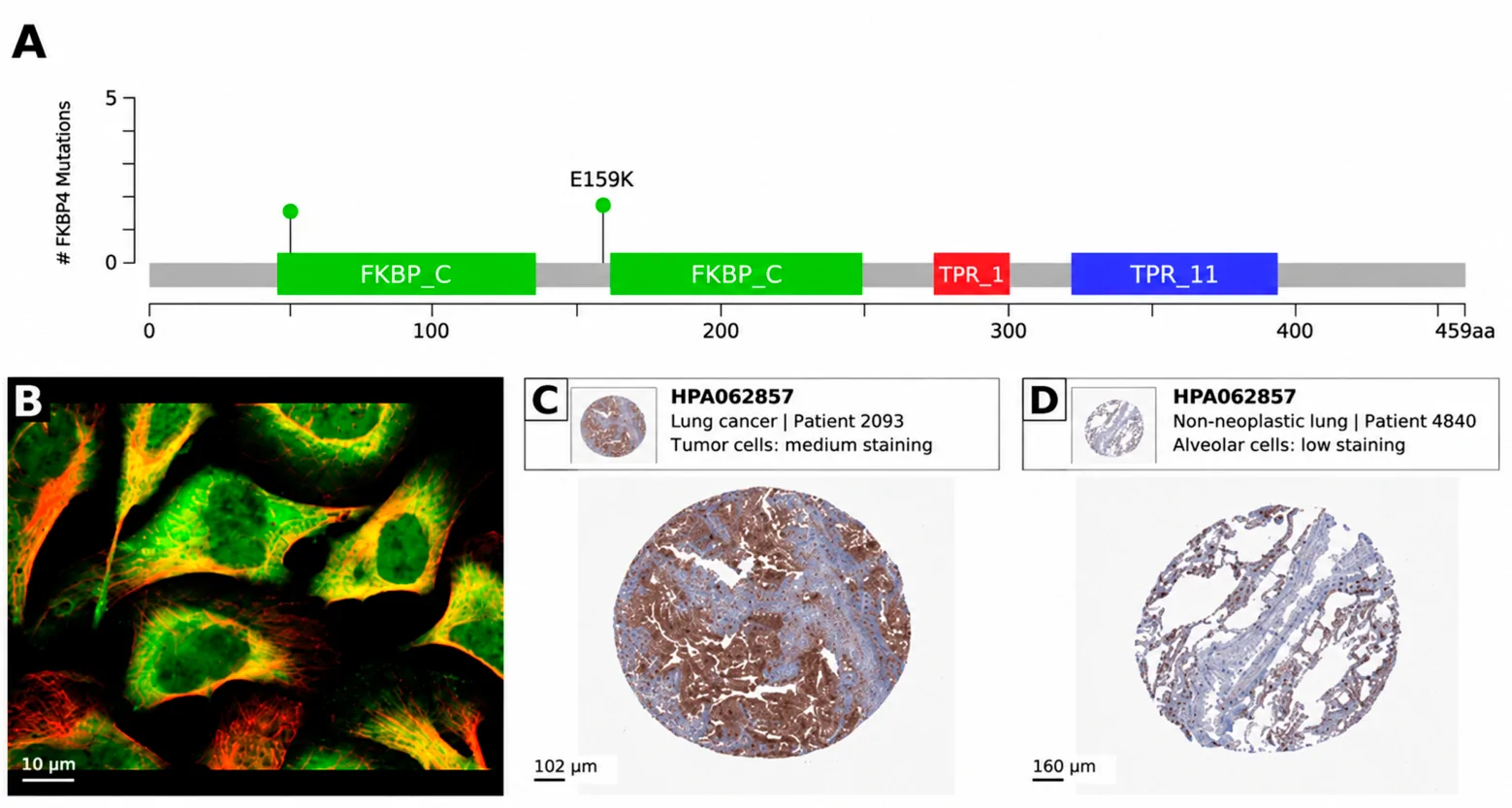
Public genomic and protein-localization context for FKBP4. (**A**) cBioPortal protein-domain view of two coding alterations in the TCGA-LUAD PanCancer Atlas mutation profile. E159K is labeled; no recurrent hotspot was observed. (**B**) HPA immunofluorescence from U2OS cells using HPA006148. FKBP4 is green and microtubules are red; scale bar, 10 μm. (**C**) HPA lung adenocarcinoma image using HPA062857 (patient 2003). Tumor staining was annotated as medium, with moderate intensity in more than 75% of cells; scale bar, 100 μm. (**D**) HPA non-neoplastic lung image using HPA062857 (patient 4840). Alveolar-cell staining was annotated as low; scale bar, 100 μm. Panels (**B**–**D**) are representative and unpaired. They were reviewed descriptively and were not quantitatively compared. Image credit: Human Protein Atlas. HPA images are reproduced under the Creative Commons Attribution 4.0 International License.

The cBioPortal profile contained two *FKBP4* coding alterations among 511 tumors (0.39%; Figure 2A). E159K was documented, and no recurrent hotspot was apparent. HPA immunofluorescence showed cytosolic and nucleoplasmic FKBP4 signal in U2OS cells (Figure 2B). Representative HPA images showed FKBP4 staining in lung adenocarcinoma and non-neoplastic lung tissue (Figure 2C,D). These images provide descriptive protein context and do not support tumor–normal quantification.

### 3.3. No CellMiner Association Remains Significant After FDR Correction

**Figure 3:**
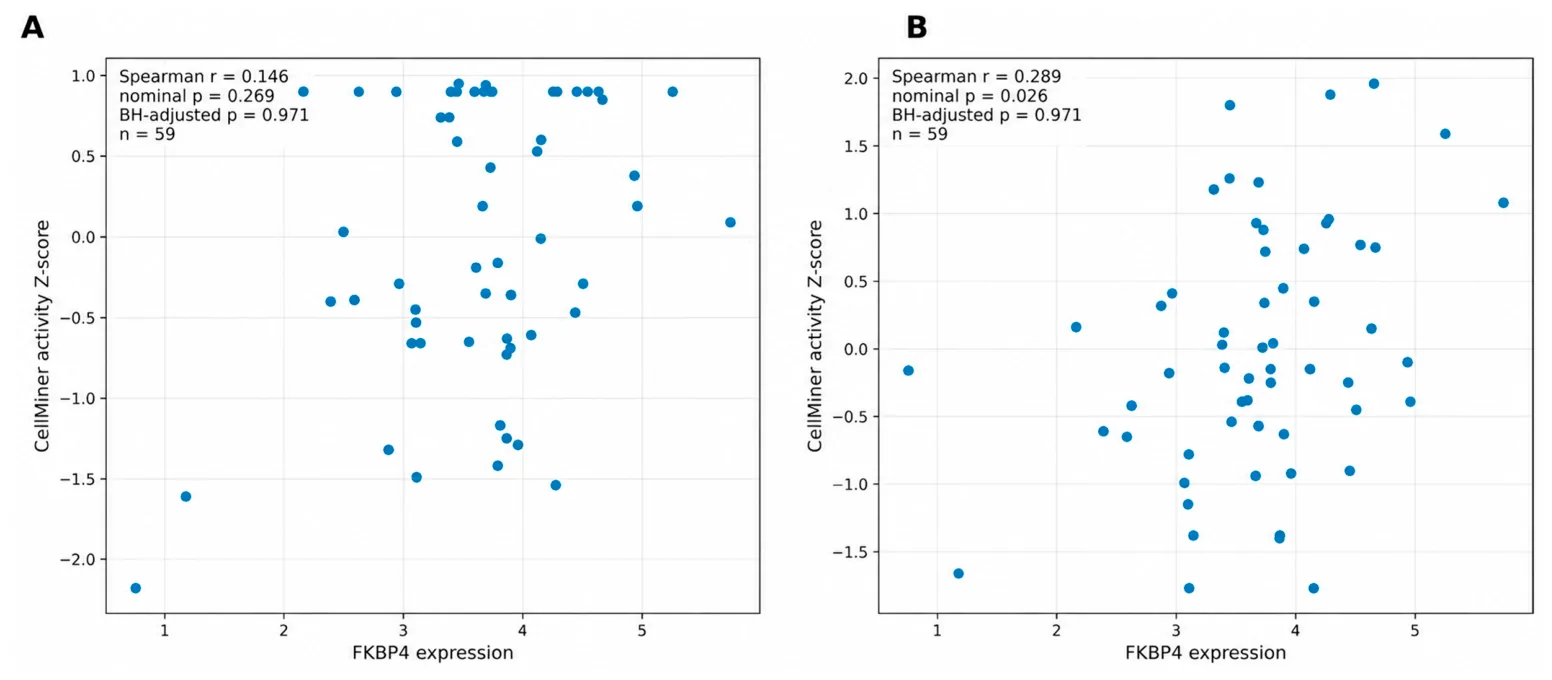
CellMiner associations retained from the original submission. (**A**) *FKBP4* expression and 5-fluorodeoxyuridine 10-mer activity across 59 NCI-60 cell lines. (**B**) *FKBP4* expression and cladribine activity across 59 NCI-60 cell lines. Spearman r, nominal p, BH-adjusted p, and n are shown. Neither association remained significant after FDR correction. Dataset S2 provides all 263 results.

Seven of 263 CellMiner activity profiles had nominal *p* < 0.05, but none remained significant after Benjamini–Hochberg correction (minimum adjusted *p* = 0.971). Cladribine showed r = 0.289, nominal *p* = 0.026, and adjusted *p* = 0.971. The 5-fluorodeoxyuridine 10-mer profile showed r = 0.146, nominal *p* = 0.269, and adjusted *p* = 0.971. Thus, only cladribine was nominally significant, and neither profile met the adjusted threshold. Table 3 lists these two profiles. Figure 3 shows both associations as scatter plots. Dataset S2 provides all 263 results.

### 3.4. FKBP4 Transcript Knockdown and CCK-8 Measurements in H1299 Cells

**Figure 4:**
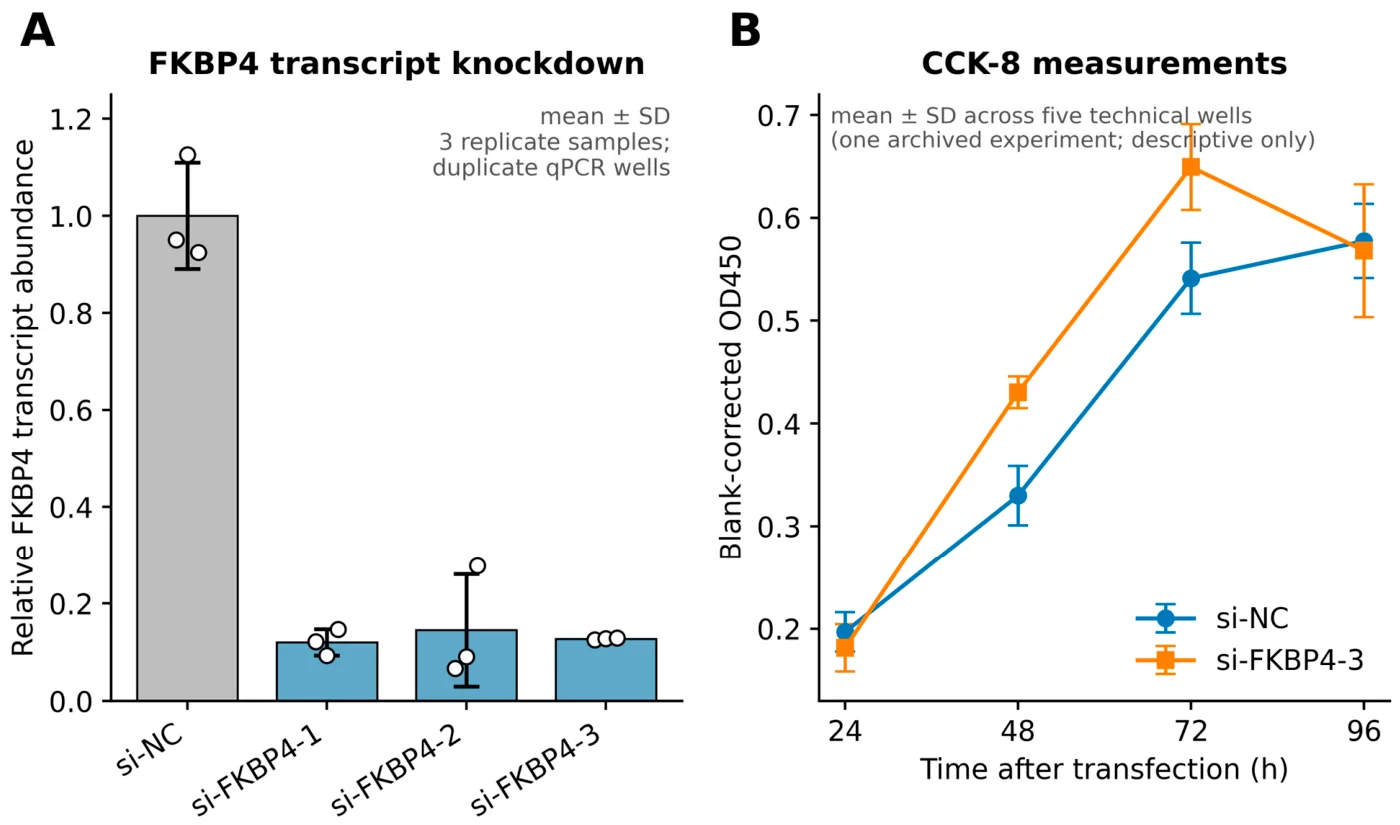
FKBP4 transcript knockdown and CCK-8 measurements in H1299 cells. (**A**) Relative FKBP4 transcript abundance after three targeting siRNAs, normalized to si-NC. Values are mean ± SD for three replicate samples, each measured in duplicate qPCR wells; separate transfections could not be confirmed. (**B**) Blank-corrected CCK-8 OD450 after si-FKBP4-3 or si-NC. Values are mean ± SD across five technical wells per condition and time point from one archived experiment; the CCK-8 result is descriptive. No protein-level knockdown measurement or orthogonal functional assay accompanied panel (**B**); it does not define a cellular phenotype.

Relative *FKBP4* transcript abundance was 0.120 ± 0.027, 0.145 ± 0.116, and 0.127 ± 0.002 after si-FKBP4-1, si-FKBP4-2, and si-FKBP4-3, respectively (Figure 4A). These values corresponded to reductions of 88.0%, 85.5%, and 87.3%. Only si-FKBP4-3 was evaluated in the archived CCK-8 experiment. The available qPCR records did not establish that these replicate samples originated from independent transfections.

At 48 h, mean blank-corrected OD450 was 0.430 ± 0.015 with si-FKBP4-3 and 0.330 ± 0.029 with si-NC. Corresponding values at 72 h were 0.649 ± 0.042 and 0.541 ± 0.035. At 24 h, the values were 0.181 ± 0.023 and 0.197 ± 0.019, respectively. At 96 h, they were 0.568 ± 0.065 and 0.577 ± 0.036. Because these values came from technical wells in one experiment, they are reported descriptively without inferential testing. No protein-level FKBP4 measurement or orthogonal functional readout accompanied this archived CCK-8 experiment.

## 4. Discussion

*FKBP4* showed tumor co-expression patterns that passed set-wise correction, whereas the pharmacogenomic and cellular findings were more limited. The displayed apoptosis and peroxisome correlations remained significant after conservative multiple-testing correction. No CellMiner profile remained significant after FDR control. In one archived H1299 experiment, mean CCK-8 values were higher at 48 and 72 h after si-FKBP4-3 transfection.

The bidirectional apoptosis pattern argues against a uniform pro- or anti-apoptotic state across LUAD tumors. It may instead reflect heterogeneous cellular programs within bulk tissue. These co-expression associations do not establish FKBP4-mediated regulation of apoptosis. Tumor purity, cellular composition, and proliferation-related covariates were not adjusted, and no independent cohort was used for validation. FKBP4 is an HSP90 co-chaperone involved in client maturation and trafficking [5,6,7]. The present study did not measure HSP90 clients or apoptosis directly.

Peroxisomes regulate lipid metabolism and redox homeostasis, which are relevant to tumor biology [24,25,26]. *FKBP4* correlated with peroxisome genes in both positive and negative directions. This pattern may reflect shared tumor states rather than direct FKBP4 control. The same bulk-tissue, covariate, and external-validation limitations apply to these associations.

The cBioPortal profile showed no recurrent *FKBP4* hotspot. Only two coding alterations were observed among 511 tumors. These data provide genomic context but do not identify a recurrent mutational driver. The HPA panels provide localization context only. They do not establish differential protein abundance or regulatory mechanisms.

No drug association remained significant after FDR correction in the CellMiner screen. Thus, this dataset does not support *FKBP4* as a broad NCI-60 drug-sensitivity marker. The multi-lineage composition of NCI-60 further limits LUAD-specific inference.

CCK-8 measures WST-8 reduction and is influenced by both cell number and reducing capacity [27]. Therefore, the higher mean OD450 values at 48 and 72 h cannot establish proliferation, survival, or another defined phenotype. They also came from five technical wells in one archived experiment, so reproducibility across independent experiments is unknown. FKBP4 protein depletion was not assessed during the CCK-8 window. Only si-FKBP4-3 was tested, so sequence-specific and off-target effects cannot be excluded. A second siRNA phenotype, siRNA pool, rescue experiment, and orthogonal functional assays were unavailable. Accordingly, this observation should not be treated as functional validation of an FKBP4-dependent phenotype.

The direction of the descriptive H1299 CCK-8 observation differs from reports in PC9, H1975, and A549 cells [11,28,29]. Those studies linked *FKBP4* depletion to reduced growth or other malignant phenotypes. However, without independent replication or orthogonal validation, this difference should not be interpreted as evidence of context-dependent FKBP4 biology. Genotype, FKBP4 dependence, transfection conditions, assay design, and timing remain possible explanations that require direct multi-model testing.

Several limitations define the scope of this exploratory study. TCGA analysis used one bulk-tumor cohort without external validation or adjustment for tumor purity, cellular composition, or proliferation-related covariates. CellMiner also spans multiple tumor lineages. The cell-based evidence derives from archived experiments in which independent biological replication could not be confirmed. CCK-8 was the only functional readout, only si-FKBP4-3 was tested in that assay, and FKBP4 depletion was not verified at the protein level during the assay window. These constraints limit assignment of a reproducible FKBP4-dependent phenotype but do not negate the value of the corrected tumor-level associations or the preliminary cellular observation as hypothesis-generating evidence. The present analysis therefore helps define specific questions for follow-up rather than establishing mechanism. Future studies should include independent biological replication, protein-level confirmation of *FKBP4* knockdown, and orthogonal assays such as direct cell counting, EdU incorporation, colony formation, apoptosis analysis, or cell-cycle analysis. Additional siRNAs or pooled siRNAs, rescue experiments, and validation in additional appropriately characterized LUAD models would further test FKBP4 dependence.

Taken together, the set-wise corrected TCGA results identify statistically supported within-cohort co-expression patterns associated with *FKBP4*, whereas the FDR-corrected CellMiner analysis does not support a broad drug-response association. The H1299 result remains preliminary and model-specific. Viewed together, these findings refine the current evidence base by separating statistically supported tumor-level associations from observations that require functional confirmation. This distinction provides a focused basis for future mechanistic studies.

## 5. Conclusions

*FKBP4* expression showed statistically supported co-expression associations with apoptosis- and peroxisome-related genes in TCGA-LUAD after set-wise correction. These associations identify tumor-level transcriptional patterns but do not establish FKBP4-mediated pathway regulation. The CellMiner screen identified no FDR-significant drug association. The H1299 CCK-8 result represents a preliminary cellular observation from one archived experiment and cannot define a specific phenotype or confirm FKBP4 dependence. Overall, this exploratory study clarifies the relative strength of transcriptomic, genomic, pharmacogenomic, and cellular evidence for FKBP4 in LUAD and identifies testable priorities for independent functional validation.

## Figures and Tables

**Table 1:** Small interfering RNA sequences used for *FKBP4* knockdown.

Target	Strand	Sequence (5′–3′)
si-FKBP4-1	Sense	GCGUGCUGAAGGUCAUCAAtt
si-FKBP4-1	Antisense	UUGAUGACCUUCAGCACGCtt
si-FKBP4-2	Sense	UGGAGUUGUUUGAGUUUAAtt
si-FKBP4-2	Antisense	UUAAACUCAAACAACUCCAtt
si-FKBP4-3	Sense	GGAAGGUAAAUACAAGCAAtt
si-FKBP4-3	Antisense	UUGCUUGUAUUUACCUUCCtt
si-NC	Sense	UUCUCCGAACGUGUCACGUtt
si-NC	Antisense	ACGUGACACGUUCGGAGAAtt

**Table 2:** Primer sequences used for RT-qPCR.

Target	Primer	Sequence (5′–3′)
FKBP4	Forward	CAGAAAGCACAGGCCCTTCG
FKBP4	Reverse	AGAGGCCCTTCTCGTTGT
GAPDH	Forward	GATTCCACCCATGGCAAATT
GAPDH	Reverse	CTGGAAGATGGTGATGGGATT

**Table 3:** CellMiner activity profiles retained from the original submission and shown in Figure 3.

Compound	Spearman r	Nominal *p*-Value	BH-Adjusted *p*-Value
5-Fluorodeoxyuridine 10-mer	0.146	0.269	0.971
Cladribine	0.289	0.026	0.971

## Data Availability

Source data for the cell experiments are provided in the accompanying minimal dataset. Public datasets were obtained from TCGA-LUAD, cBioPortal, the Human Protein Atlas, CellMiner, and MSigDB. Additional processed data are available from the corresponding authors upon reasonable request.

## Supplementary Materials

The following supporting information can be downloaded at https://www.mdpi.com/article/10.3390/biology15181661/s1. Supplementary Table S1: FKBP4 correlations with MSigDB Hallmark genes in TCGA–LUAD; Dataset S1: FKBP4 H1299 qPCR and CCK-8 source data and processed summaries; Dataset S2: CellMiner 263-profile Spearman analysis with nominal and adjusted *p*-values.
